# Designing information provision to serve as a reminder of altruistic benefits: A case study of the risks of air pollution caused by industrialization

**DOI:** 10.1371/journal.pone.0227024

**Published:** 2020-01-16

**Authors:** Hidenori Komatsu, Hiromi Kubota, Nobuyuki Tanaka, Hirotada Ohashi

**Affiliations:** 1 Energy Innovation Center, Central Research Institute of Electric Power Industry, Kanagawa, Japan; 2 Environmental Science Research Laboratory, Central Research Institute of Electric Power Industry, Abiko-shi, Chiba, Japan; 3 School of Engineering, The University of Tokyo, Bunkyo-ku, Tokyo, Japan; Middlesex University, UNITED KINGDOM

## Abstract

A well-known phenomenon is that humans perceive risks to threaten future generations as more dangerous in many cases. However, this tendency could be changed depending on certain conditions and could potentially be explained by the evolution of altruism. Our multi-agent simulation model, which was constructed to identify attributes contributing to subjective assessment of a risk source based on kin selection theory, showed that support from relatives can affect the agents’ subjective risk assessment. We utilize this insight, which has never been explored in the context of nudge, to show that real-world messages reminding respondents that they are supported by their relatives can moderate the perception of a risk source as extremely dangerous. A randomized control trial based on an internet questionnaire survey was conducted to identify the intervention effect of such messages, using air pollution caused by industrialization as the risk source for the case study. Our analysis suggests that messages moderate extreme attitudes. Presentation of additional visual information can boost the sense of familial support and increase the effect of a message compared with a message comprising only textual information. The attributes and personality traits of the respondents who are responsive to the intervention message are also discussed.

## Introduction

Nudge is defined as “any aspect of the choice architecture that alters people’s behavior in a predictable way without forbidding any options or significantly changing their economic incentives.” In other words, it stimulates intuitive decision-making to help people make better choices [1]. A wide variety of applications have been successful [2], and several categories of nudges have been proposed. A well-known example is MINDSPACE, which stands for Messenger, Incentives, Norms, Defaults, Salience, Priming, Affect, Commitment, and Ego. For effective information provision, it is important to consider salience, which is the basis for the phenomenon that human attention is drawn to what is novel and seems relevant [3], which eventually influences behavior [4]. Some techniques based on loss aversion [5] or anchoring [6] have been proposed as useful tools, but it is not yet fully understood which conditions lead to the perception of novelty and relevance. The concept of nudge emphasizes humans’ irrational decision-making. However, evolutionary perspectives could reveal rationality at a deeper level within human decision-making which has been traditionally been regarded as an anomaly [7]. Such perspectives could potentially provide a consistent methodology for designing more salient information provision to stimulate intuitive responses, which may be a product of human evolution [8, 9].

One example of human intuitive decision-making that should be considered in information provision is risk judgment. Previous studies have sought to explain how humans respond to risks in terms of evolutionary adaptations [10, 11]. The responses to risks are specific to evolutionary domains [12]. These findings, based on evolutionary rationality, could explain some observations that cannot be explained by conventional concepts of rationality or selfishness. For example, responses to risk can be influenced by the effect on other people [13], which could be explained as an evolutionary adaptation based on social interactions.

Assuming that evolutionary psychology can explain how intuitive decision-making has evolved, the process of adaptation is essentially a loop of human reproduction and death, which cannot be demonstrated experimentally. To overcome this limitation, simulation models are useful tools for understanding the adaptation and basic foundations for investigating what kind of messages are effective for information provision.

Common evolutionary simulation models to identify the origin of responses to risks are expanded versions of evolutionary game theory [14], which was originally used to explain the evolution of cooperation among non-kin individuals based on reciprocal altruism [15], although there are approaches other than evolutionary game theory [16]. There has been no analysis using mathematical or simulation models to discuss whether the evolution of altruism affects the response to life-threatening risks. Thus, we have previously constructed simulation models focusing on kin selection [17], hypothesizing that the subjective assessment of a risk source could be influenced by the evolution of altruism when the risk is perceived to threaten future generations [18]. The model suggested that those who are supported by their relatives recognize a risk source as safer, even if the population has evolved to recognize it as dangerous on average.

Focusing on this insight, in this work, we design messages to remind respondents that we are supported by our ancestors in our everyday life and ascertain whether the messages moderate the respondents’ extreme attitudes to perceiving a risk source as dangerous in the real world. Previously, we conducted a preliminary experiment for similar information provision, which partly pre-specified the intervention effect for the present study. In the experiment, the control group received simple textual information describing the risks of the air pollution and the benefits of industrialization. The main target group received textual messages as an intervention highlighting support from past generations and to future generations along with the simple information that the control group received. Here, we expected that recognition of industrialization as benefitting future generations would promote respondents’ perceived need to incur less costs themselves and indirectly bring about effects similar to those brought about by the perception that they were supported by relatives. The intervention for the main target group increased the sense of being supported by relatives and moderated attitudes to perceive air pollution as dangerous, but the effect was not statistically significant [19]. Descriptions in the designed message of the relationship among generations, of who benefits whom and how, may have been complicated to present using only text, which may have decreased information acceptance. In the present study, we aimed to increase the intervention effect by presenting an additional illustration to make the structure for giving and receiving benefits clear and increase the sense of being supported. We used information provision about air pollution caused by industrialization as a case study. We found that presenting additional visual information boosted the sense of familial support and the effect of the message, compared with a message comprising only textual information. These results showed that we achieved the aim of our study.

The remainder of this paper is organized as follows. In Section 2, we describe the settings of the experiments. In Sections 3 and 4 we describe and discuss the results of the experiments, and in Section 5 we provide concluding remarks, including future work.

## Method

### Survey overview

We conducted an internet-based questionnaire survey to ascertain the intervention effects of information provision. We designed the overall framework and the questionnaires for the experiment, although we used a survey company to distribute the questionnaires to respondents and collect the results. The respondents were registered with the company. We performed a quantitative analysis using a randomized control trial (RCT) and a qualitative analysis using an open-ended question, based on the samples that we received from the survey company. The survey company clearly declared in their terms and conditions of membership, with which all the respondents agreed, that the questionnaire results would be used for research purpose. The questionnaire survey was totally anonymized, did not retrieve personal information, did not use samples taken from human bodies, and assumed no psychological distress of the respondents.

The samples were obtained on November 29th and 30th, 2018, from respondents who were over 20 years old and living in Japan. The sample’s basic attributes, including age and sex, were based on the information that the survey company had on record. The samples were collected from equal numbers of male and female respondents. We excluded respondents with number of children who are living with the respondents or working in paid jobs was larger than the number of children. The total number of valid samples was 2764. The sex ratio was eventually 100.3 due to the exclusion of invalid samples, which was higher than that in the general population in Japan of 94.8 [20].

**Fig 1:**
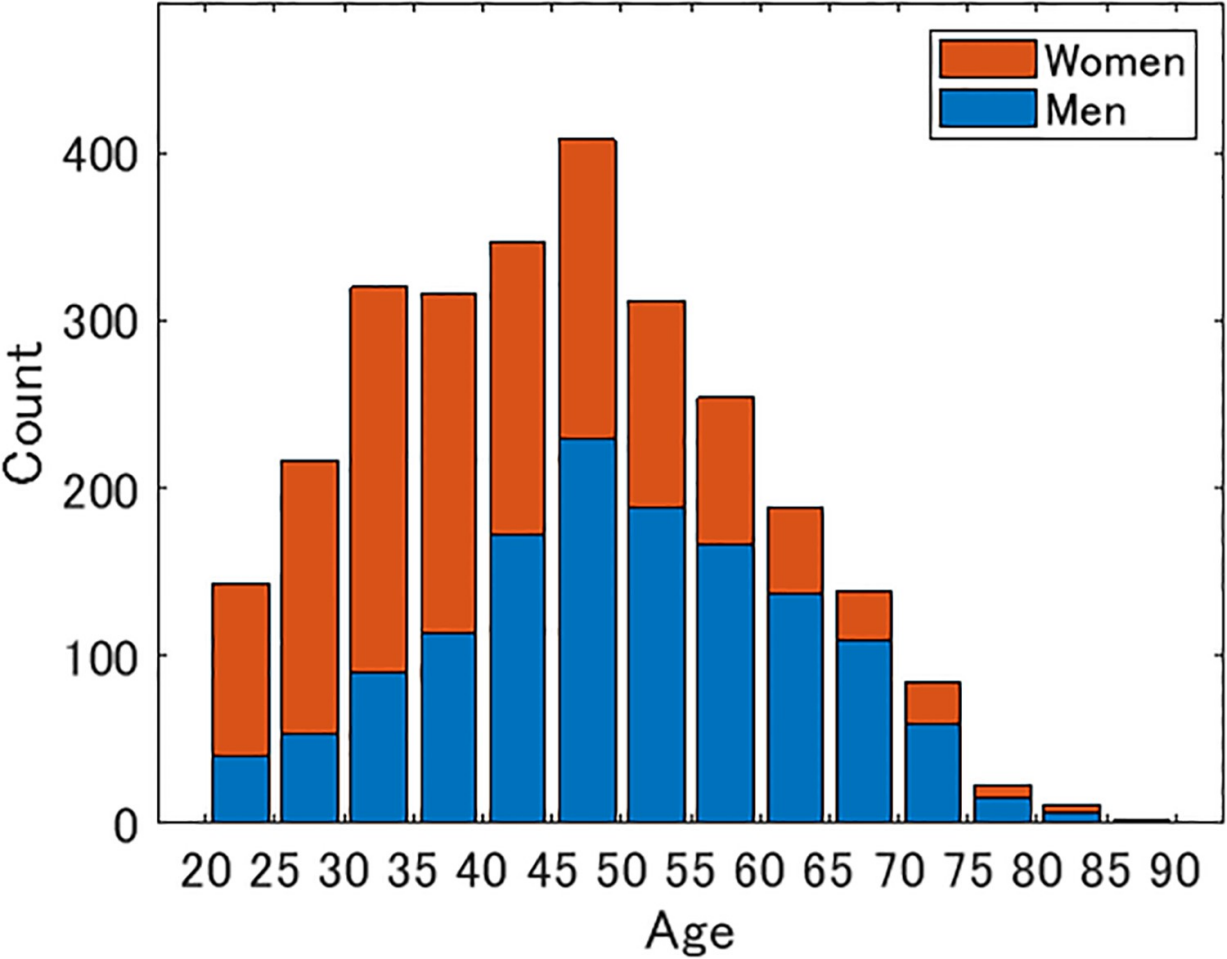
Number of respondents by age and sex.

The average age of all the respondents was 45.3. The average age of the male respondents was 49.7 and that of the female respondents was 41.0. The number of male respondents was largest in the 45–50 year old age group, whereas the number of female respondents was largest in the 30–35 year old age group (Fig 1).

Japan is divided into nine regions. From north to south, the regions are Hokkaido, Tohoku, Kanto, Chubu, Kinki, Chugoku, Shikoku, Kyushu, and Okinawa. Because the samples were collected randomly and independent of the region where the respondents live, the sample ratios by region are equivalent to the census data (Table 1). The mean numbers of children that the respondents have, that are living with the respondents, and that are working were 1.1, 0.7, and 0.4, respectively (Table 2). Other attributes relevant to the respondents’ parents are shown in Table 3.

**Table 1:** Sample ratios by region.

Region	Collected samples		Percentage from census (%)^※^
	Percentage (%)	Counts	
**Hokkaido**	5.4	148	4.7
**Tohoku**	5.3	147	6.6
**Kanto**	37.6	1040	36.8
**Chubu**	16.3	450	15.0
**Kinki**	19.1	528	16.5
**Chugoku**	5.2	144	5.9
**Shikoku**	2.8	78	3.1
**Kyushu**	7.6	210	10.4
**Okinawa**	0.7	19	1.1

^※^Retrieved from the 2019 data at https://www.stat.go.jp/data/kakei/setai_bunpu.html.

Regions are listed from north to south.

**Table 2:** Characteristics of respondents’ children.

Number of children		Percentage (%)	Counts
**Mean number**	0	42.8	1182
	1	17.3	477
	2	29.8	823
	3	8.6	239
	4	1.1	30
	5 or more	0.5	13
	1.1		
**Number of children living with respondent**			
**Mean number**	0	54.7	1513
	1	21.8	602
	2	18.3	505
	3	4.4	121
	4	0.7	20
	5 or more	0.1	3
	0.7		
**Number of children who are working**			
**Mean number**	0	78.0	2155
	1	9.4	261
	2	10.0	277
	3	2.3	64
	4	0.2	5
	5 or more	0.1	2
	0.4		

**Table 3:** Characteristics of respondents’ parents.

Living with parents in the same house or at the same site	Percentage (%)	Counts
With neither parent	77.3	2136
With either parent	9.4	260
With both parents	13.3	368
**Parents working in paid jobs**		
Neither parent	67.9	1877
Either parent	17.1	473
Both parents	15.0	414

### Survey design for intervention

**Fig 2:**
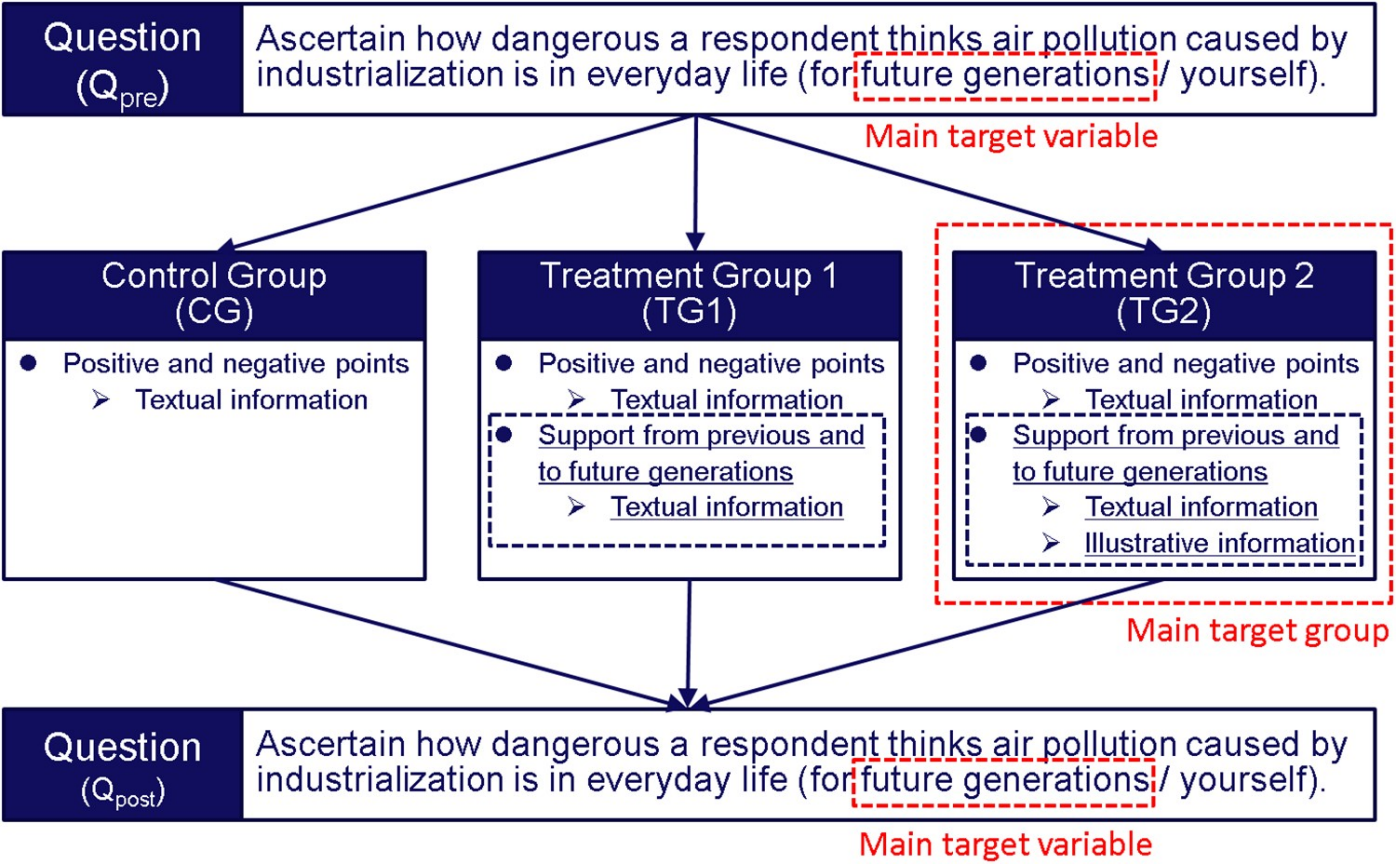
Flowchart of the experimental procedures. Black dashed lines indicate interventions. Red dashed lines indicate the main target of the interventions.

Using internet-based questionnaires, an RCT was conducted to ascertain whether the designed messages could moderate attitudes to recognize air pollution as dangerous. Fig 2 shows the flowchart of the experimental procedures.

**Fig 3:**
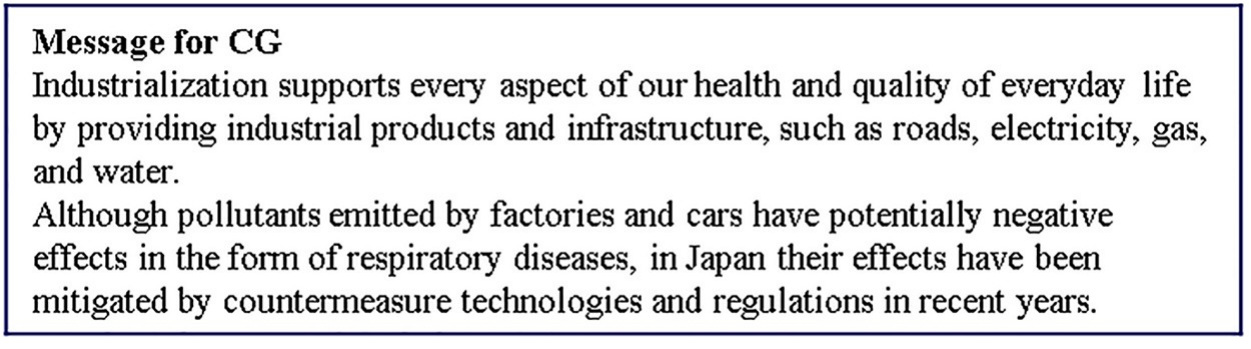
Messages presented to the CG group.

**Fig 4:**
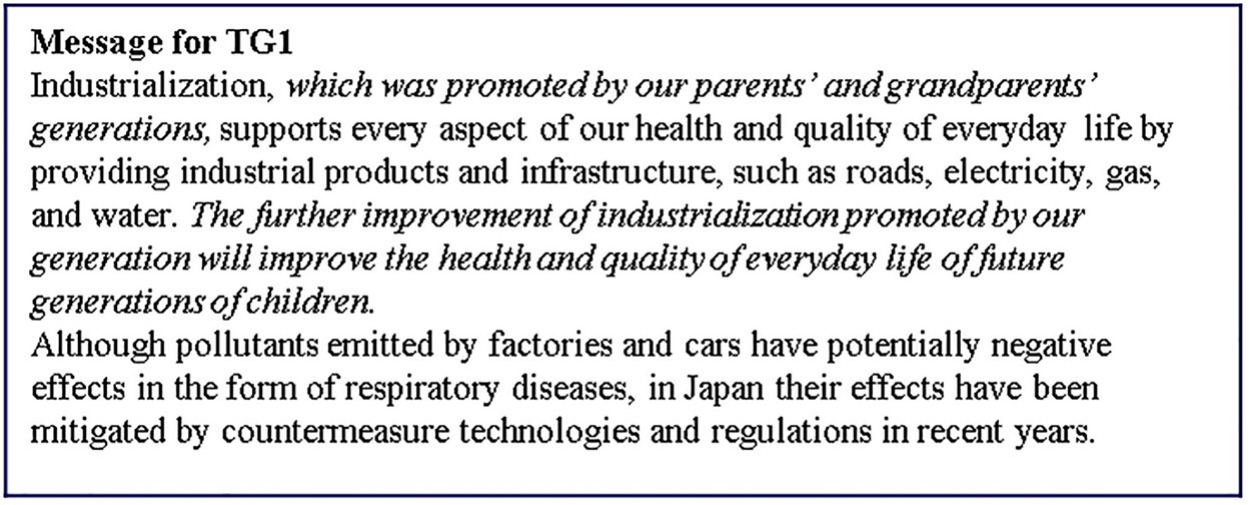
Messages presented to the TG1 group. Italic parts in the text are the intervention messages.

**Fig 5:**
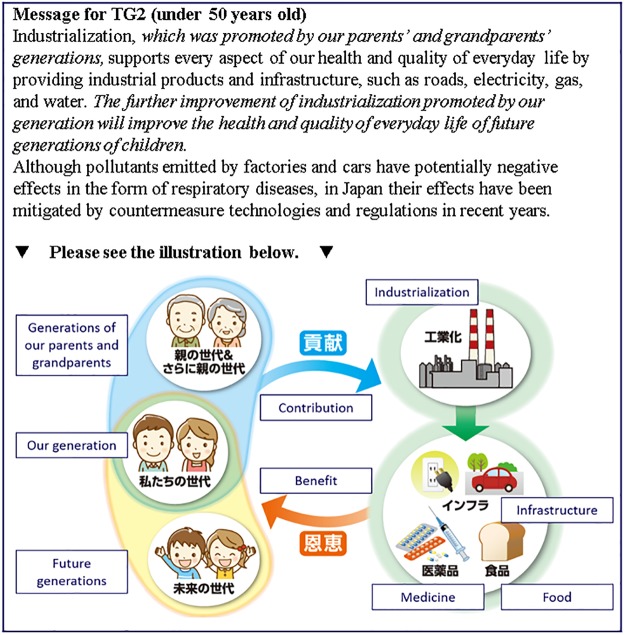
Messages presented to the TG2 group (under 50 years old). Italic parts in the text are the intervention messages.

**Fig 6:**
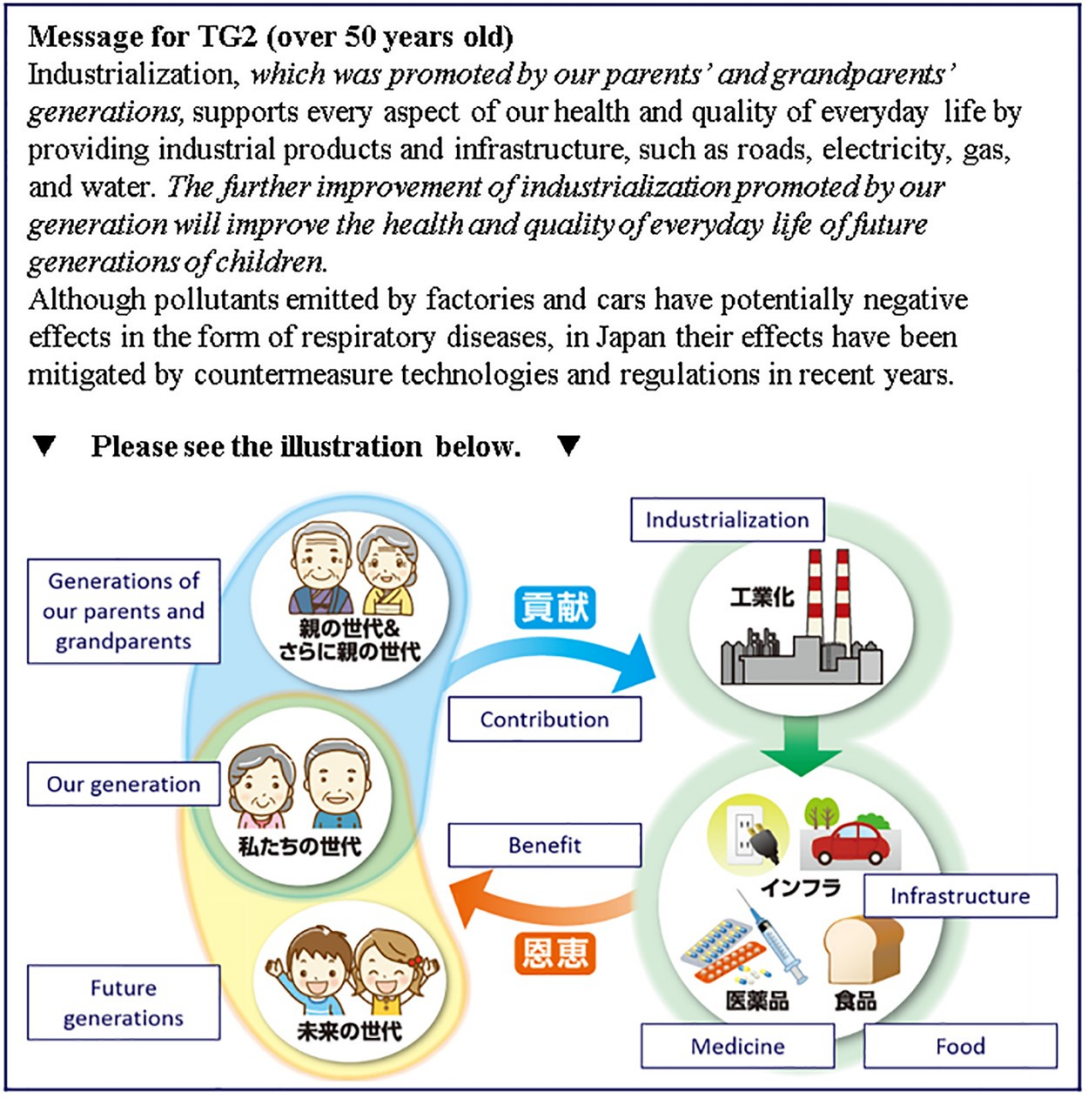
Messages presented to the TG2 group (over 50 years old). Italic parts in the text are the intervention messages.

In the questionnaires, we first asked respondents to think how dangerous or safe air pollution caused by industrialization is in daily life (*Q*_pre_) for future generations (Future generations) and for the respondents themselves (Yourself). Then we assigned the respondents by simple randomization to one of three groups that received either simple information only about the risks of the air pollution and the benefits of the industrialization (Control Group; CG) as a control, or one of two types of designed messages (Treatment Groups 1 and 2; TG1 and TG2) as an intervention (Figs 2–6 and Table 4).

**Table 4:** Group definitions in the interventions.

Provided information	Presentation type	CG	TG1	TG2 (Main target group)
Positive and negative points of industrialization	Textual	✓	✓	✓
Support from previous generations and to future generations	Textual		✓	✓
	Illustrative			✓
**Number of samples**		921	924	919

CG: Control Group receiving only the information about the risks of the air pollution and the benefits of the industrialization, TG1: Treatment Group 1 receiving additional sentences about support from previous generations and to future generations, TG2: Treatment Group 2 receiving an additional illustration highlighting the structure of support from previous generations and to future generations. ✓ Indicates the information was provided.

CG received the most basic textual information highlighting the positive and negative points of industrialization. We designed this message to be as fair as possible for the readers by clarifying both positive points and negative points of industrialization. The positive points are that industrialization supports daily life with industrial products and infrastructure, whereas the negative points are that industrialization generates air pollutants that can cause respiratory diseases, although the amount of pollutants in Japan has been decreased by countermeasures and regulations in recent years.

TG1 received two additional sentences combined with the simple textual information that CG received. One of the additional sentences suggests that we are supported by previous generations through industrialization, and the other sentence suggests that future generations will be supported through the industrialization of the present generation. The latter additional message is designed so that the respondents perceive that they are not alone in supporting future generations, and it was expected that this would increase the sense of being supported by previous generations.

TG2 received the same textual message as TG1, with an additional illustration highlighting the structure of how contributions to industrialization by previous generations and by the present generation benefit present generations and future generations in the form of products. Considering the wide variety of respondents’ ages, the appearance of the previous generations and the present generation was switched depending on whether they were under or over 50 years old. Many respondents over 50 years old were not expected to have living parents. Thus, for respondents over 50 years old, we presented old people in traditional old-style clothes as “generations of parents and grandparents.” Immediately after presenting the intervention messages, we again asked the respondents in all three groups the same question as shown to the respondents before the interventions (*Q*_post_). Attitudes toward air pollution caused by industrialization were measured on a 5-point Likert scale for both *Q*_pre_ and *Q*_post_ (see the “Questionnaire” section).

The design of the interventions was based on our previous preliminary survey that investigated the three types of messages consisting of only textual information. In that survey we compared the messages highlighting support from past generations, support of future generations, and the combination of support from past generations and of future generations as the richest information [19]. The richest textual information, which corresponded to TG1 in this work, showed the largest intervention effect in the survey, yet the intervention effect was still not enough to be statistically significant. TG2 was the main target group in this work and the message was designed to complement the drawback of TG1, that textual information alone is complicated to understand and accept. The additional illustration as visual information was expected to communicate how the benefits of industrialization are generated and given, to reinforce the sense of being supported by previous generations, and to boost the intervention effect compared with TG1.

## Results

### Before interventions

This section presents the status of the samples before the intervention. The questions to be investigated in this section are as follows:

- Were the samples sufficiently randomized?
- Was air pollution a risk source that was perceived as more dangerous to future generations than to the respondents themselves?
- How different were the attitudes toward air pollution by sex, age, and region?

**Fig 7:**
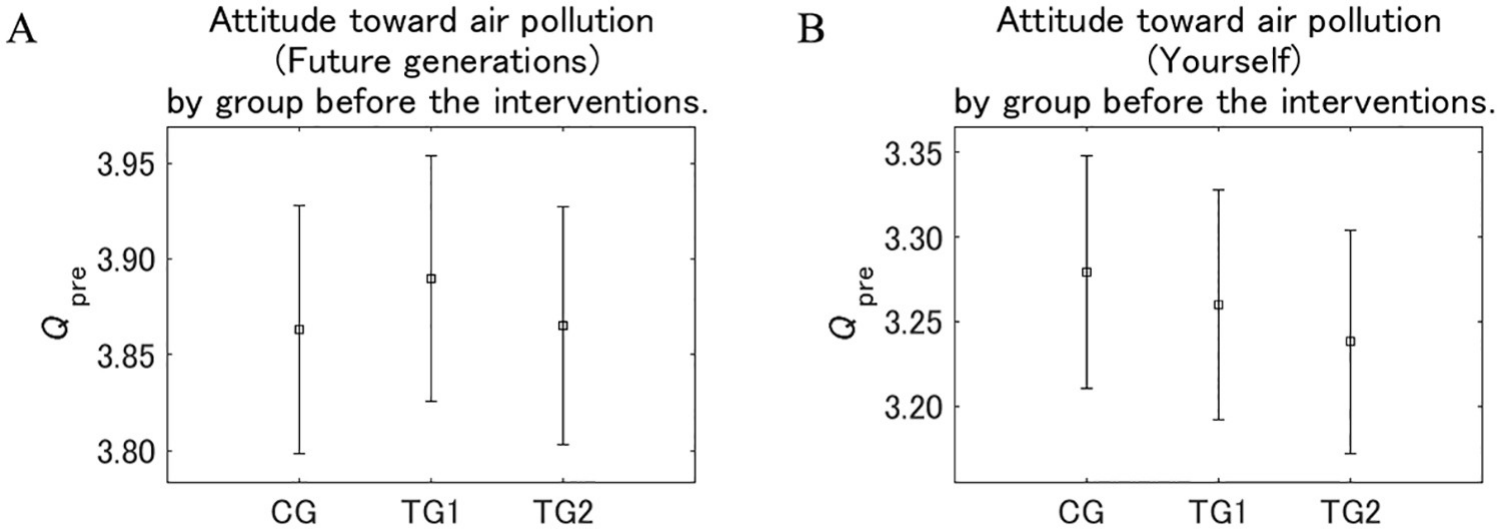
Attitude toward air pollution by group before the interventions. The value on the vertical axis is higher the more dangerous pollution is perceived to be. Error bars show 95% confidence intervals.

The attitude toward the effect of air pollution on future generations and the respondents themselves before they received the interventions (responses to *Q*_pre)_ are shown by group in Fig 7. ANOVAs and comparisons of attitudes were conducted for future generations (Future generations) (Tables 5 and 6) and the respondents themselves (Yourself) (Tables 7 and 8). The attitudes were not significantly different among the CG, TG1, and TG2 groups, both for future generations and the respondents themselves, suggesting that the samples were sufficiently randomized. Within the same group, the value for future generations was consistently higher than for the respondents themselves, suggesting that the effect of air pollution is recognized as more dangerous to future generations than to the respondents themselves.

**Table 5:** One-way analysis of variance for attitudes toward air pollution before the interventions (*Q*_pre_) for future generations (future generations).

Source	SS	df	MS	F	probability > F
**Group**	0.401	2	0.200	0.208	0.813
**Error**	2666.771	2761	0.966		
**Total**	2667.172	2763			

SS: sum-of-squares, df: degrees of freedom, MS: mean squares, F: F ratio

**Table 6:** Comparison of attitudes toward air pollution before the interventions (*Q*_pre_) for future generations (future generations).

Compared groups		Lower end of 95% confidence interval	Estimated mean	Upper end of 95% confidence interval	*p*
CG	TG1	−0.083	0.025	0.132	0.854
CG	TG2	−0.081	0.026	0.134	0.832
TG1	TG2	−0.106	0.002	0.109	0.999

**Table 7:** One-way analysis of variance for attitudes toward air pollution before the interventions (*Q*_pre_) for the respondents themselves (Yourself).

Source	SS	df	MS	F	probability > F
**Group**	0.764	2	0.382	0.351	0.704
**Error**	3009.760	2761	1.090		
**Total**	3010.524	2763			

SS: sum-of-squares, df: degrees of freedom, MS: mean squares, F: F ratio

**Table 8:** Comparison of attitudes toward air pollution before the interventions (*Q*_pre_) for the respondents themselves (Yourself).

Compared groups		Lower end of 95% confidence interval	Estimated mean	Upper end of 95% confidence interval	*p*
CG	TG1	−0.093	0.021	0.135	0.898
CG	TG2	−0.133	−0.019	0.095	0.917
TG1	TG2	−0.155	−0.041	0.073	0.680

**Fig 8:**
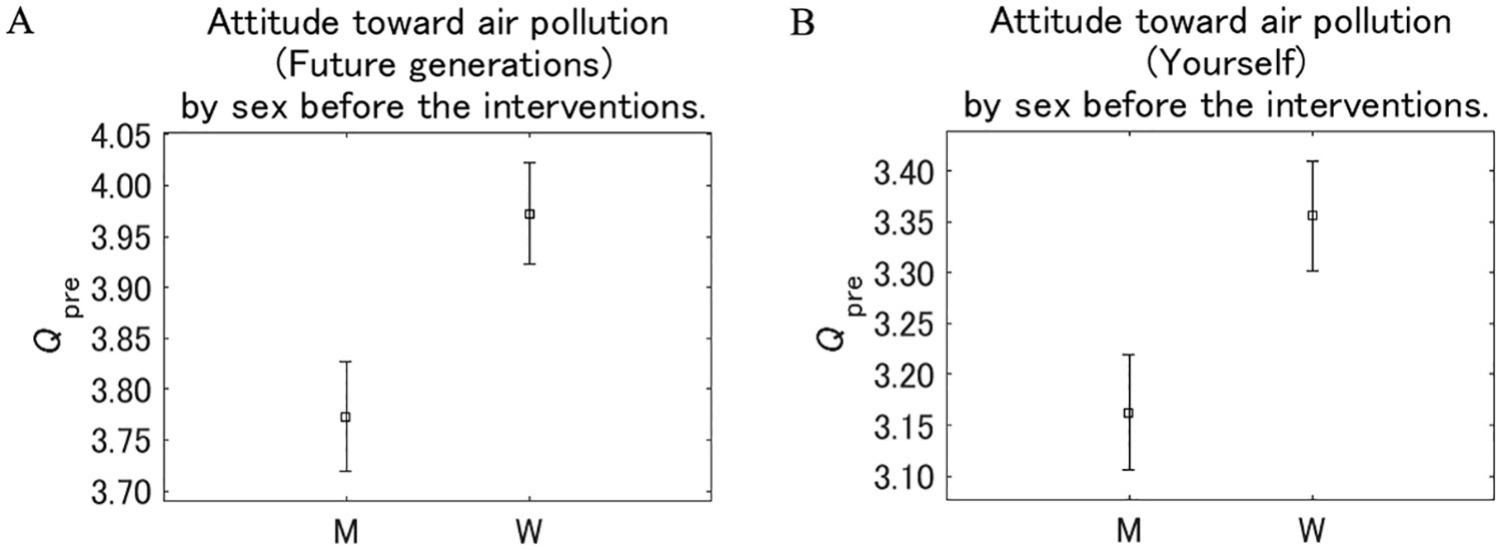
Attitude toward air pollution by sex before the interventions. M: men, W: women. The value on the vertical axis is higher the more dangerous pollution is perceived to be. Error bars show 95% confidence intervals.

Fig 8 shows the attitude toward the effect of air pollution on future generations and the respondents themselves before they received the interventions (responses to *Q*_pre_) according to sex. For future generations and the respondents themselves, women recognized air pollution as more dangerous than men. Within the same sex, the value for future generations was consistently higher than for the respondents themselves, as the same with by group shown in Fig 7.

**Fig 9:**
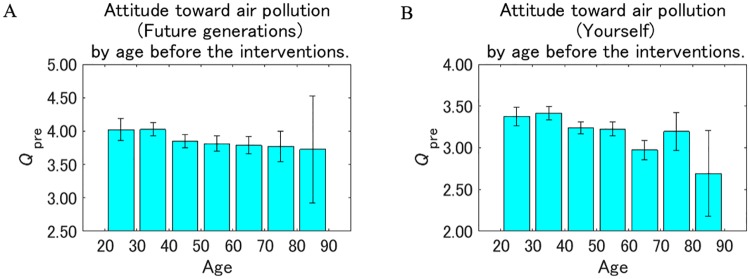
Attitude toward air pollution by age before the interventions. The value on the vertical axis is higher the more dangerous pollution is perceived to be. Error bars show 95% confidence intervals.

Fig 9 shows the attitude toward the effect of air pollution on future generations and the respondents themselves before they receive the interventions (responses to *Q*_pre_) by age. For both future generations and the respondents themselves, younger respondents tended to recognize air pollution as more dangerous than the older respondents, although the trend was not consistent for the 30–40 and 70–80 year old age groups for the respondents themselves. Within the same age, the value for future generations was consistently higher than for the respondents themselves, suggesting that the respondents recognized the effect of air pollution as more dangerous to future generations than to the respondents themselves, independent of their ages.

**Fig 10:**
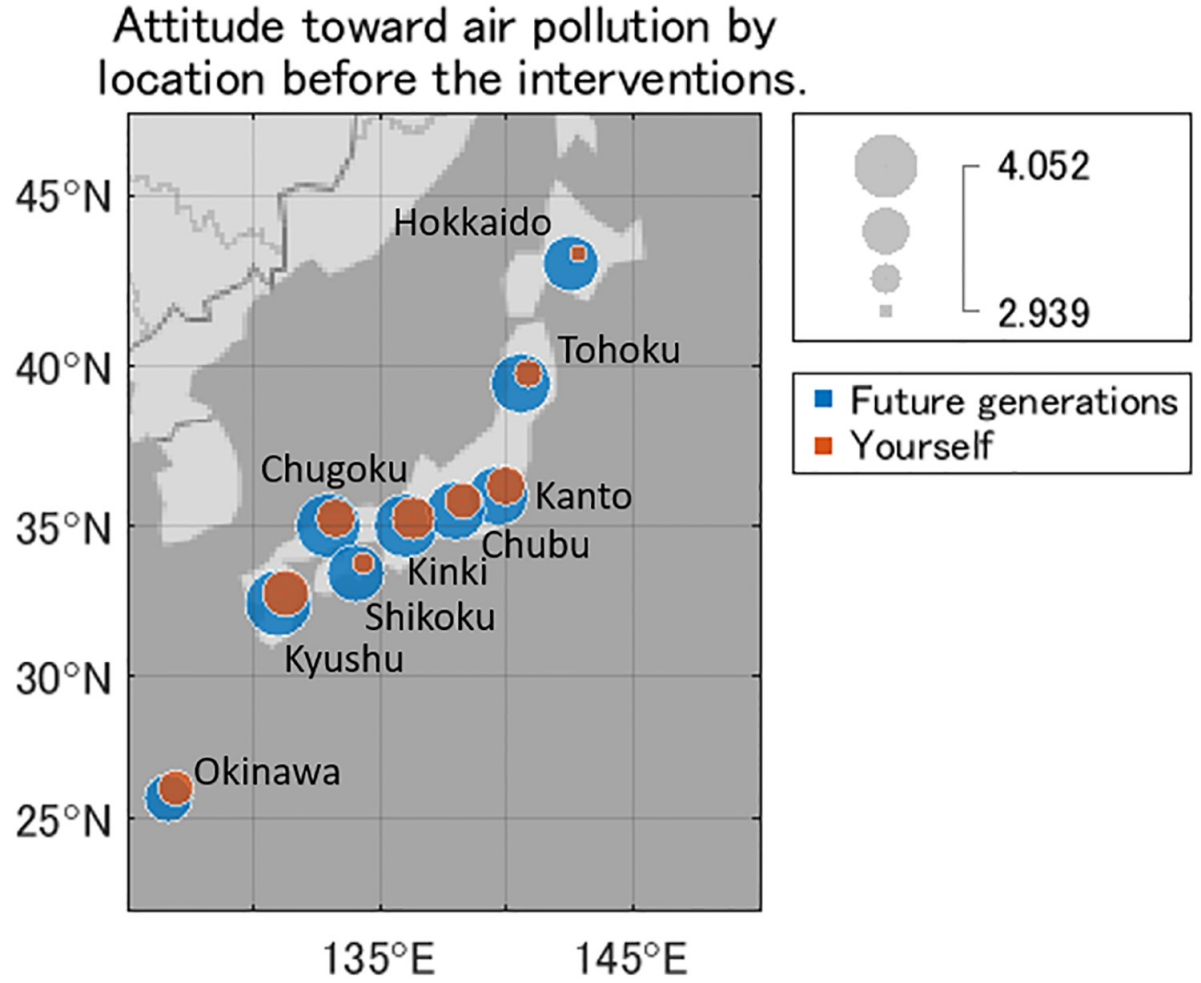
Attitude toward air pollution by location before the interventions. Blue represents the attitude toward future generations, and orange represents the attitude toward the respondents themselves. The radius is larger the more dangerous air pollution is perceived to be.

The average attitudes toward air pollution before the interventions are plotted by area on a map of Japan (Fig 10). The effect of air pollution on future generations was recognized as more dangerous than the effect on the respondents themselves. This trend was consistent in all nine areas. The attitudes toward future generations were similar for all areas. The difference in attitudes between the effect on future generations and the respondents themselves was the smallest in Okinawa and the effect on future generations was perceived to be low compared with the other areas. However, these trends are not statistically significant because Okinawa had the smallest number of samples (Table 1).

Although our questionnaires did not mention any specific pollutants, such as PM_2.5_, and used the general wording ‘air pollution’, PM_2.5_ is the most widely recognized air pollutant in everyday life in Japan because there are television programs that forecast air quality and focus on PM_2.5_. Thus, the respondents may have been thinking about PM_2.5_ when they read about air pollution in our questionnaires. The effect on the respondents themselves was perceived to be more dangerous in areas where the actual levels of PM_2.5_ are high [21]. A typical example of this is Kyushu.

### Overall intervention effects

This section investigates the effects of the interventions by treatment group. The questions to be investigated in this section are as follows:

- Was the intervention effect of TG2 for future generations (main target group and variable) larger than that of CG?
- Was the intervention effect of TG2 for the respondent themselves (Yourself), instead of future generations (Future generations), still larger than that of CG?
- Did the additional illustrative information in TG2 improve the intervention effect compared with the textual information in TG1?
- Supposing the intervention effects were TG2 > TG1 > CG, were the perceptions of being supported by older relatives and of supporting younger relatives in the order TG2 > TG1 > CG, too?

**Fig 11:**
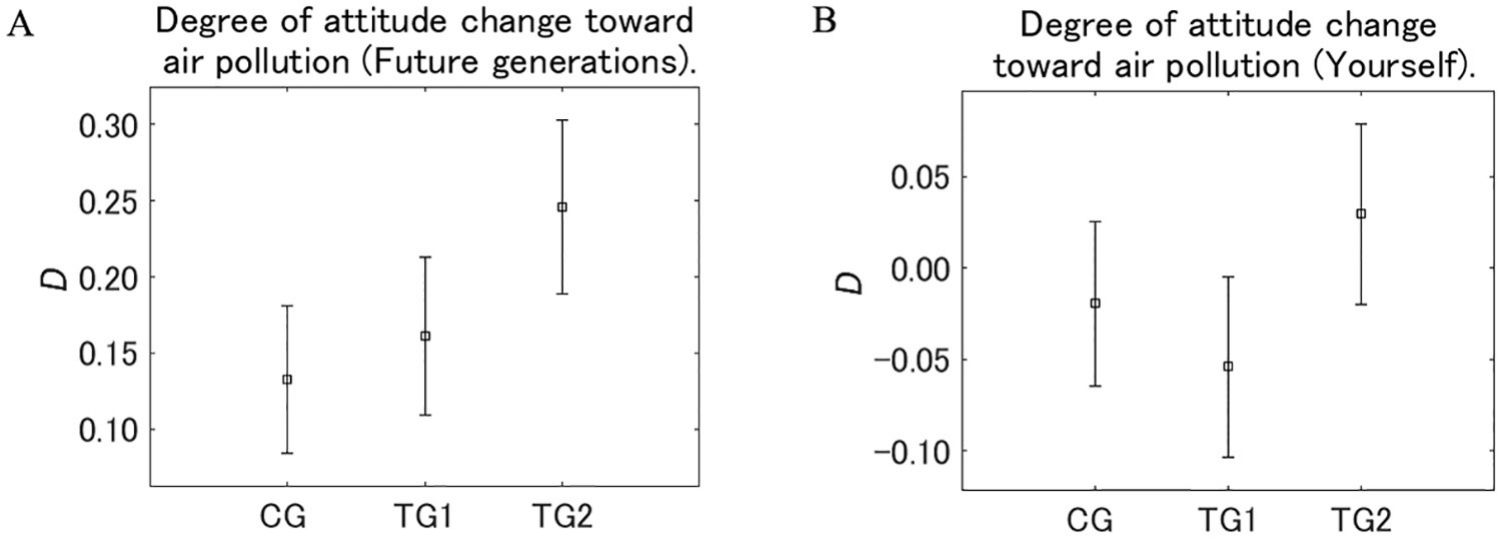
Degree of attitude change toward air pollution after receiving one of the designed messages (*D*). The value on the vertical axis is higher the less dangerous the perception of air pollution changed to be. Error bars show 95% confidence intervals.

We defined the degree of the attitude change toward the air pollution after receiving one of the designed messages as *D*, which is the difference between the answers to *Q*_post_ and *Q*_pre_ for every sample. Fig 11 shows *D* for future generations and the respondents themselves by group. For future generations, *D* for all the groups was positive and the averages were TG2 > TG1 > CG. *D* for TG2 was larger than for TG1 (*p* < 0.05) and CG (*p* < 0.01). These results suggest that the combination of additional text and the illustration for TG2 increased *D* compared with the control group CG, and the additional illustration for TG2 also increased *D* compared with TG1, for which only additional textual messages were used.

For the respondents themselves, *D* for TG2 was significantly larger than that for TG1 (*p* < 0.05), although the difference between TG2 and CG was not significant. Considering that *D* of TG2 for the respondents themselves was not positive significantly, and even significantly negative in TG1, the message for TG2 may not affect the respondents themselves. However, at least the combination of the additional messages and the supportive illustration showed a significant effect on the attitude toward future generations, and the effect on the respondents themselves was better than for presenting only the textual information in TG1.

**Fig 12:**
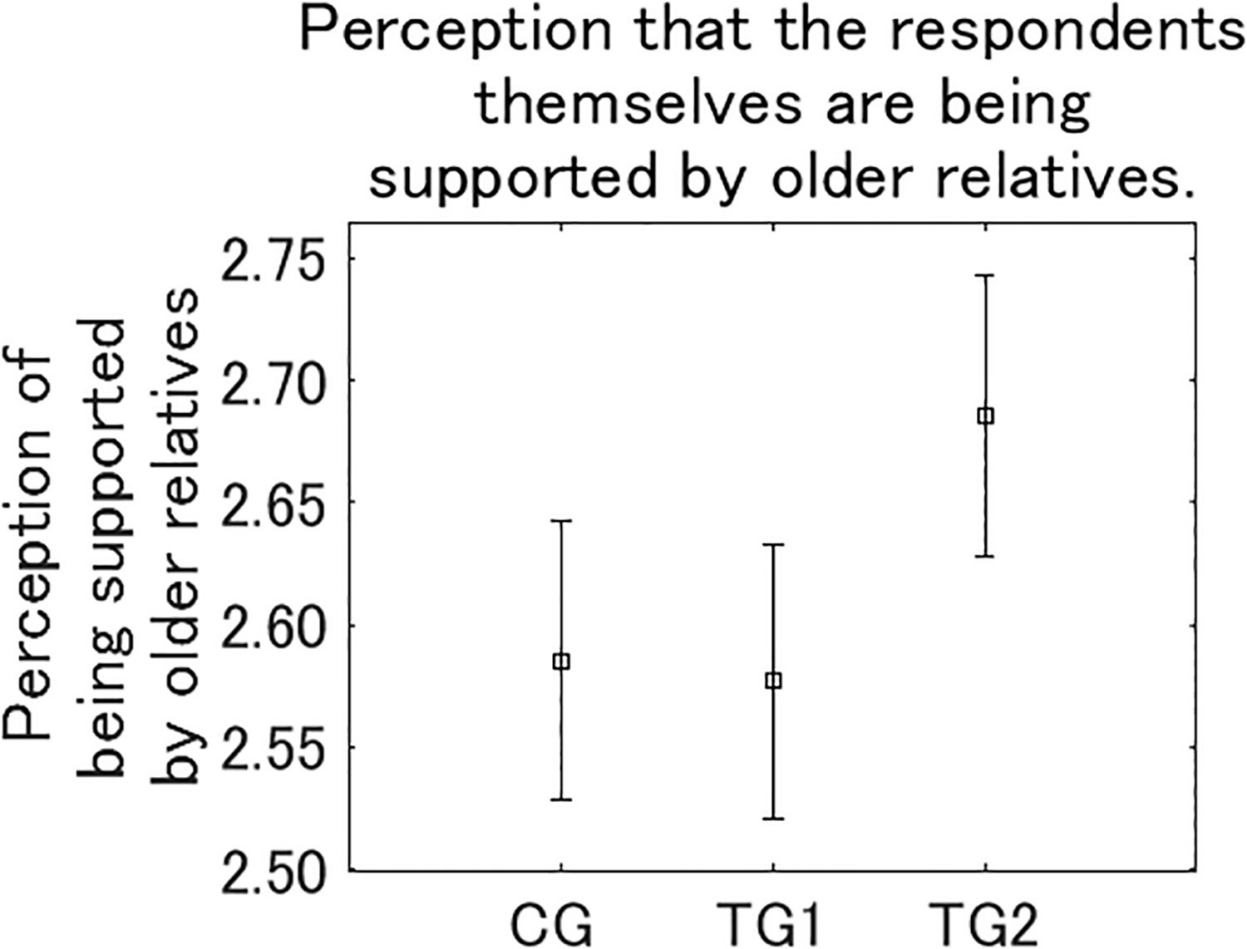
Perception that the respondents themselves are being supported by relatives belonging to older generations. Error bars show 95% confidence intervals.

**Fig 13:**
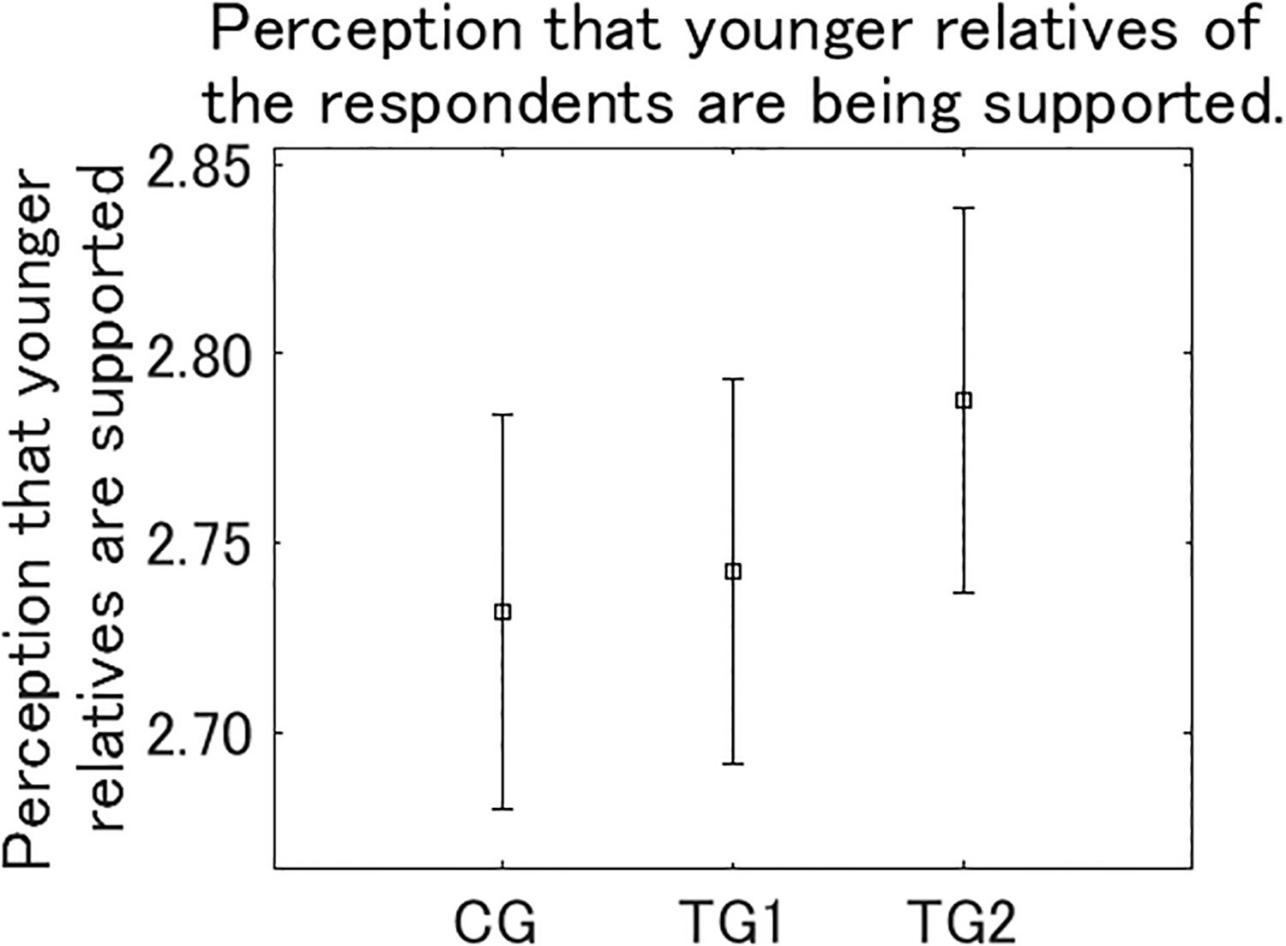
Perception that younger relatives of the respondents are being supported by industrialization. Error bars show 95% confidence intervals.

After *Q*_post_, we asked respondents how much they feel their health and quality of everyday life are being supported by relatives who belong to older generations, including their parents and grandparents, on reading the presented information (Fig 12). The perceptions of being supported was the largest in TG2, and significantly larger than in CG (*p* < 0.05) and TG1 (*p* < 0.01). After the question to ascertain the perception of support by older relatives, we asked respondents how much they feel that industrialization is supporting health and quality of everyday life of their younger relatives, including their children and grandchildren, on reading the presented information (Fig 13). The perceptions that younger relatives are being supported was TG2 > TG1 > CG, although the differences between the three groups were not significant. These results suggest that the intervention in TG2 increases the perception of being supported by older relatives more than the other interventions, although its effect to increase the perception that younger relatives are being supported is relatively weak.

To investigate how the intervention effect that moderates the perception of air pollution as dangerous is related to the perception of being supported by older relatives and the perception that the younger generations are being supported, we conducted correlation analysis (Tables 9 and 10). Aggregating all the samples in the three groups, *D* for future generations (Future generations) were significantly correlated with both the perception of being supported by older relatives (*p* < 0.001) and the perception that younger relatives were being supported (*p* < 0.001). *D* for the respondents themselves (Yourself) was also significantly correlated with both the perception of being supported by older relatives (*p* < 0.001) and the perception that younger relatives were being supported (*p* < 0.05), but the correlation coefficients were smaller than for future generations. Although the correlation coefficients by group were not consistently significant due to the relatively small sample sizes compared with the whole sample, they were consistently positive. These results suggest that when the perception of being supported by older relatives and the perception that younger relatives are being supported are increased, the feeling that air pollution is dangerous could be moderated.

**Table 9:** Correlation coefficients between change in attitude (*D*) and perceptions of being supported by older relatives on receiving the intervention.

Group	Future generations	Yourself
**CG + TG1 + TG2 (*n* = 2764)**	***0.08	***0.07
**CG (*n* = 921)**	*0.08	0.05
**TG1 (*n* = 924)**	0.05	*0.07
**TG2 (*n* = 919)**	***0.11	*0.08

*, *** Difference from zero with 95% and 99.9% confidence, respectively.

**Table 10:** Correlation coefficients between change in attitude (*D*) and perceptions that younger relatives are being supported after receiving the intervention.

Group	Future generations	Yourself
**CG + TG1 + TG2 (*n* = 2764)**	***0.08	‘0.04
**CG (*n* = 921)**	***0.10	0.04
**TG1 (*n* = 924)**	*0.07	0.04
**TG2 (*n* = 919)**	*0.07	0.03

‘, *, *** Difference from zero with 90%, 95%, and 99.9% confidence, respectively.

### Intervention effects by segment

This section investigates effects of the interventions by segment. The question in this section is as follows:

- How different were the intervention effects by sex, age, and region?

**Fig 14:**
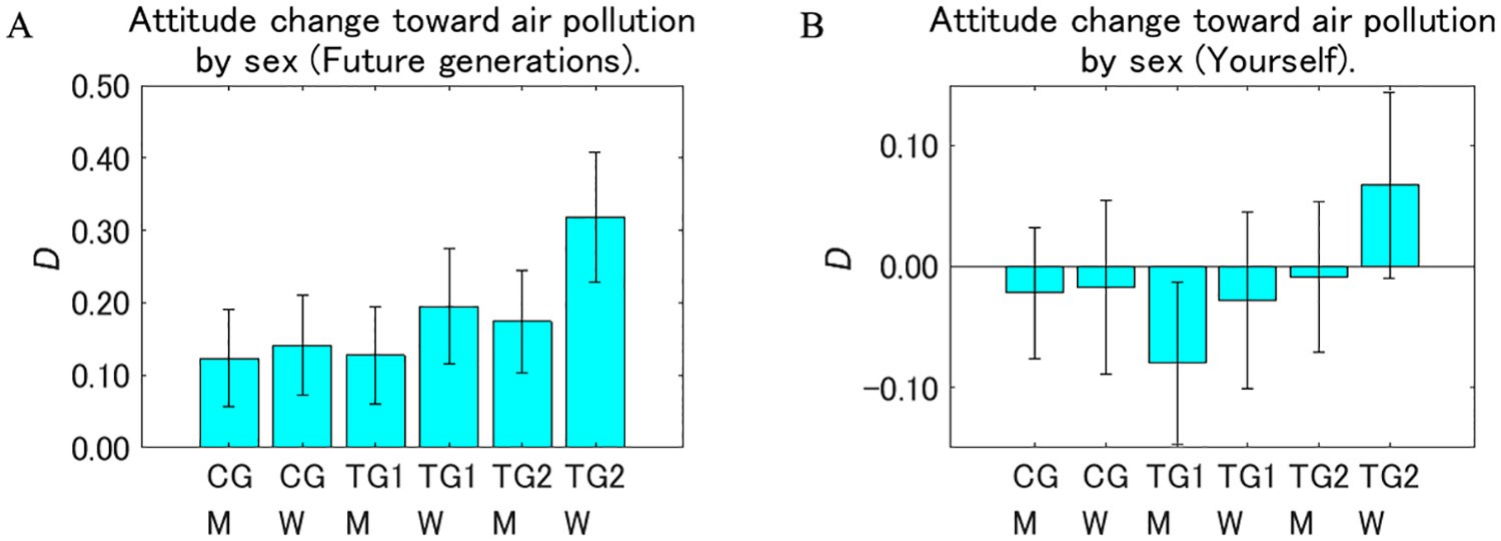
Attitude change toward air pollution after receiving a designed message (*D*) by sex. M: men, W: women. Error bars show 95% confidence intervals. The value on the vertical axis is higher the less dangerous the perception of air pollution changed to be.

We divided the samples between men and women and investigated the intervention effects by sex (Fig 14). For both future generations and the respondents themselves, female respondents showed a larger decrease in the perception that air pollution is dangerous than the male respondents in all the groups. Especially for future generations in TG2, the difference was significant (*p* < 0.05). Within the same sex, for future generations, the trend for *D* was TG2 > TG1 > CG. Over all the segments, the intervention effect was the largest for women in TG2 for future generations.

For the respondents themselves, all the other groups showed no significant increase or decrease except for the male respondents in TG1. Yet the difference between the negative *D* for men in TG1 and the positive *D* for women in TG2 was significant (*p* < 0.01), which resulted in the significant increase of the intervention effect in TG2 compared with TG1 when all the samples were aggregated (Fig 11).

**Fig 15:**
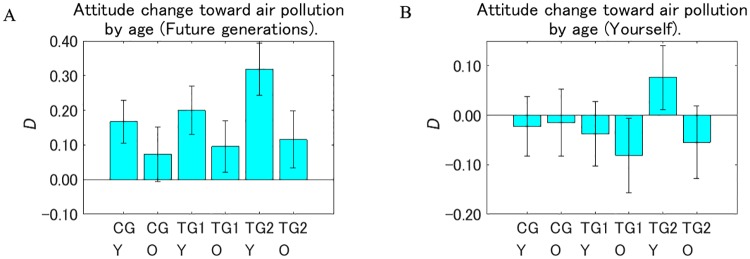
Attitude change toward air pollution after receiving a designed message (*D*) by age. Y: respondents under 50 years old, O: respondents over 50 years old. Error bars show 95% confidence intervals. The value on the vertical axis is higher the less dangerous the perception of air pollution changed to be.

Next, we divided the samples into younger and older segments to investigate the intervention effects by age (Fig 15). We defined the younger segments as being under 50 years old, and the older segments as being over 50 years old because we changed the illustration in TG2 for these age groups.

For future generations, the younger respondents showed a larger decrease in the perception that air pollution is dangerous than the older respondents in all the groups, and the differences were significant in TG1 (*p* < 0.1) and TG2 (*p* < 0.001). Within the same age segment for future generations, the trend for the intervention effects was TG2 > TG1 > CG. Over all the segments, the intervention effect was the largest in the younger segment of TG2.

For the respondents themselves, *D* of the younger respondents was significantly more positive than the negative *D* of the older respondents in TG2 (*p* < 0.01). This positive *D* of the younger respondents in TG2 was significantly more than the negative *D* of the older respondents in TG1 (*p* < 0.01), which contributed to the significant increase in the intervention effect in TG2 compared with TG1 when all the samples were aggregated within the same groups (Fig 11), similar to the intervention effects by sex.

**Fig 16:**
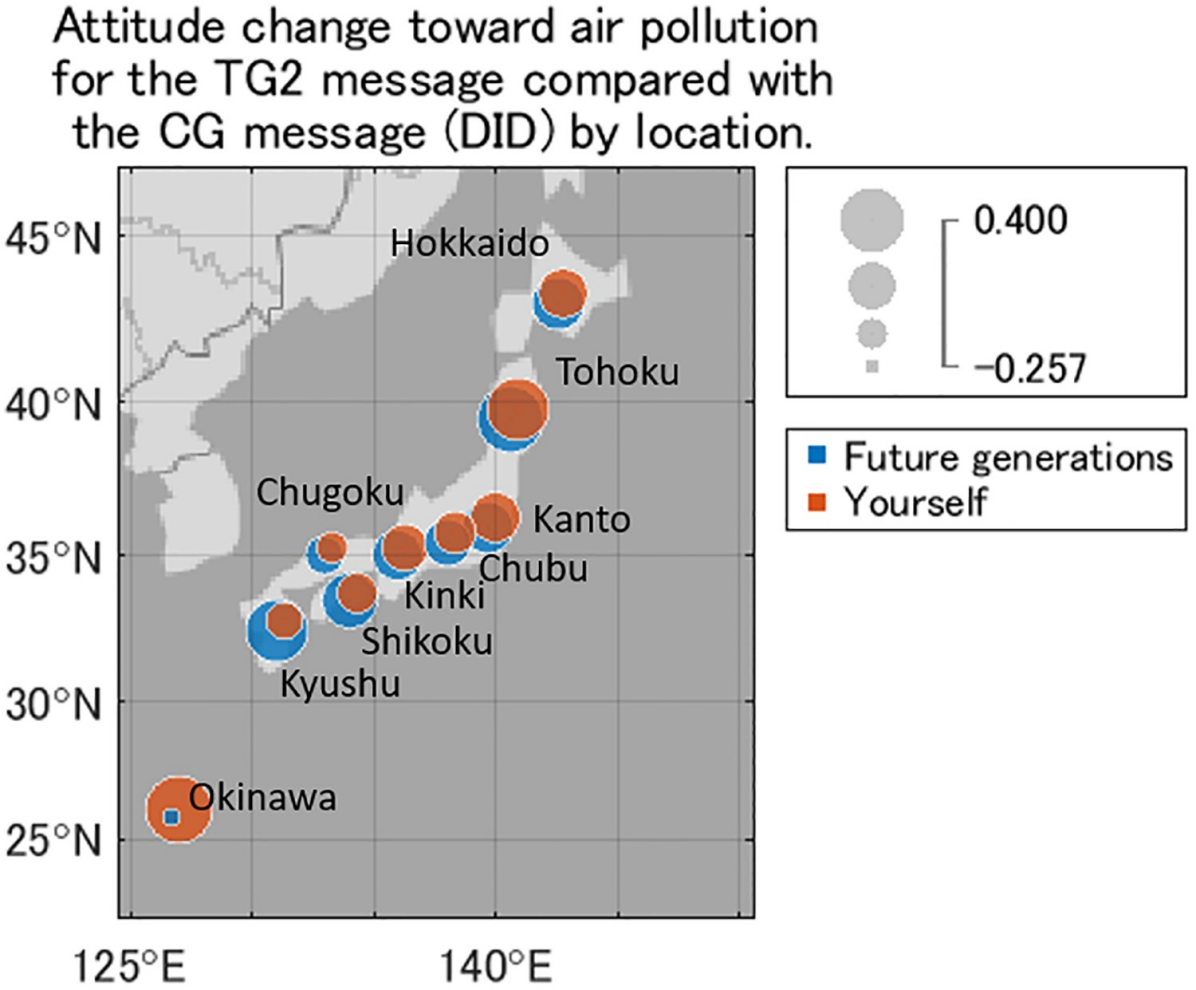
Attitude change toward air pollution for the TG2 message compared with the CG message (DID) by location. Blue represents the attitude toward future generations, and orange represents the attitude toward the respondents themselves. The radius is larger the larger the change in attitude.

Fig 16 shows the difference of *D* in TG2 compared with CG, namely the difference-in-differences (DID). In the eight areas except for Okinawa, the DID effect was consistently larger for future generations than for the respondents themselves, within the same area. For both future generations and the respondents themselves, the DID effect was larger when the air pollution was initially perceived as less dangerous, for example, in Hokkaido, Tohoku, and Shikoku. The DID effect was larger for the respondents themselves than for future generations only in Okinawa, yet there were fewer samples for Okinawa compared with the other areas.

### Panel data analysis

This section provides results of panel data analysis. The questions investigated in this section are as follows:

- How robust were the intervention effects for future generations?
- How sensitive were the attributes sex and age to the information provision?
- Which of the Big Five traits were sensitive to the information provision?

Focusing on the intervention effect in TG2 for future generations that was the largest and statistically significant compared with the control group CG, we devised a linear regression model to quantify how much the interventions moderate the perception of air pollution as dangerous. The model considered the effects of the sample attributes, including the Big Five traits, which were obtained by the Japanese version of the questionnaire template called the Ten-item Personality Inventory [22].

$$D_{f}=a_{1}\times TG1+a_{2}\times TG2+a_{3}\times S+a_{4}\times A+a_{5}\times P_{ex}+a_{6}\times P_{ag}+a_{7}\times P_{co}+a_{8}\times P_{ne}+a_{9}\times P_{op}+a_{10}$$ (1)

*TG*1, *TG*2: Target of the intervention in TG1 and TG2 respectively (0: no, 1: yes)

*S*: Sex (0: Men; 1: Women)

*A*: Age

*P*_*ex*_: Extraversion

*P*_*ag*_: Agreeableness

*P*_*co*_: Conscientiousness

*P*_*ne*_: Neuroticism

*P*_*op*_: Openness

*a*_*1*_–*a*_*9*_: Coefficients for each term

*a*_*10*_: Intercept

Here, *D*_*f*_ is the difference between the answers to *Q*_post_ and *Q*_pre_ for future generations. To determine *a*_1_–*a*_10_, we conducted forced entry regression (Table 11). *TG*2 was estimated as 0.11 (*p* < 0.01), which suggests that the intervention in TG2 increased *D*_*f*_ compared with CG and the intervention functioned as we intended. The estimates for *TG*1 were not significant, but they were positive. Comparison of the coefficients on *TG*1 and *TG*2 showed that the difference was statistically significant (*p* < 0.05). These results suggest that the intervention for TG2, in which both textual and visual information were presented, had a stronger effect than that for TG1, in which only textual information was presented. *A* was estimated as −0.005 (*p* < 0.001), suggesting that young respondents contributed significantly to *D*_*f*_, although the contribution was small. For the Big Five traits, *P*_*ag*_ was estimated as 0.015 (*p* < 0.10), suggesting that high agreeableness, which is related to altruism, contributed significantly to *D*_*f*_. The other four traits of the Big Five did not show significant contributions. *S* was estimated as 0.038, suggesting that the female segment contributed more to *D*_*f*_ compared with the male segment, yet the statistical significance was insufficient.

**Table 11:** Coefficients from linear regression analysis.

		Estimated coefficients	Standard error	*t* statistics
**Intercept**		0.229	0.163	1.405
**Intervention**	TG1	0.027	0.038	0.721
	TG2	**0.112	0.038	2.965
**Attribute variables**	Sex (Men = 0, Women = 1)	0.038	0.034	1.129
	Age	***−0.005	0.001	−3.900
**Personality variables**	Extraversion	−0.005	0.007	−0.768
	Agreeableness	‘0.015	0.008	1.863
	Conscientiousness	−0.003	0.008	−0.453
	Neuroticism	−0.004	0.008	−0.476
	Openness	0.003	0.008	0.382
**Adjusted R-squared**		0.009		
**Number of samples**		2764		

‘, **, *** difference from zero with 90%, 99%, and 99.9% confidence, respectively.

### Qualitative survey

This section provides qualitative survey results. The questions investigated in this section are as follows:

- How did the additional illustration for TG2 improve the intervention effect compared with the textual information for TG1 and CG?
- How did the respondents associate air pollution with PM_2.5_?

In the final section of the series of questions about the presented information, each respondent was asked about their impression of the messages they received by using an open-ended question. In all of the three groups, there were both positive and negative responses to this question, as well as neutral ones, such as ‘nothing special’ or ‘I have never thought of the topic’. Extracting the responses qualitatively from this section highlights examples of responses that are characteristic to each group.

In many cases in all three groups, the respondents who mentioned the positive points of industrialization changed their attitude to recognize air pollution as safer, whereas those who mentioned the negative points changed their attitude to recognize air pollution as more dangerous. For example, a positive response in CG was ‘I think that this kind of improvement should proceed and be valued as a corporate effort.’ from a 32-year-old man, who changed his attitude to recognize air pollution as safer by 1 point for both future generations and himself. A negative response in CG was ‘I was horrified to think that my parents and grandparents might have suffered huge effects from air pollution.’ from a 40-year-old woman, who changed her attitude to recognize air pollution more dangerous by 1 point for both future generations and herself. A notable neutral answer in CG was ‘I thought that we always lose something if we gain something else. It would be ideal that we do not lose anything and live freely and optimally for everything. But I know it is impossible. It is so difficult that I have no idea what I can do.’ from a 35-year-old woman, who did not change her attitude for either future generations or herself. This respondent possibly thought that there was a trade-off between industrialization and air pollution and eventually did not changed her attitude, mentioning both positive and negative points.

A response which characterizes TG1 was ‘Considering that the present-day wealth of Japan is built on the efforts of the older generations, I thought that we should try hard for future generations.’ from a 31-year-old women, who changed her attitude to recognizing air pollution as safer by 2 points for future generations and 1 point for herself. This type of response was not observed in CG, which suggests that the additional textual information for TG1 reminded the respondents of being supported by the older generations. However, the following response suggested the message for TG1 was difficult to understand: ‘It would be easier to understand with more casual presentation.’ from a 31-year-old woman, who did not change her attitudes for either future generations or herself.

In TG2, a typical response was that attitudes toward the negative points were moderated, mentioning the positive points. For example, ‘When people talk about industrialization, they tend to focus on the negative aspects, but there are also merits.’ from a 39-year-old woman, who changed her attitude to recognize air pollution as safer by 2 points for both future generations and herself, or ‘I think that people tend to respond like “There are negative effects!!” when they are asked about air pollution. I reconsidered my attitude upon reading the positive points. I am ashamed of myself thinking just in a knee-jerk way. I also felt that most people out there are responding just like that, looking at the people using an SNS (she mentioned the specific service name but we chose to use the generic term ‘SNS’ here).’ from a 22-year-old woman, who changed her attitude to recognize air pollution as safer by 2 points only for future generations. There were responses that they felt the presented message was obtrusive in highlighting either positive or negative points. A response saying that the presented message highlighted only positive points was ‘I felt pushed toward the idea that receiving benefits far outweighs the pollution. It is true that we are receiving benefits, but there should not be only good points. I feel anxious at the same time.’ from a 67-year-old man, who changed to recognize air pollution as safer by 1 point only for future generations. There was an opposite response of ‘The questionnaire is encouraging anxiety.’ from a 53-year-old man, who did not change his attitudes for future generations or himself.

For all the samples over the three groups, 23 respondents out of 2764 mentioned ‘PM_2.5_’ or ‘PM something’ in the open-ended question, nevertheless the designed messages mentioned only ‘air pollution’, and did not name a specific air pollutant. This suggests that a certain proportion of respondents associated air pollution with PM_2.5_ on reading the presented information. This is consistent with respondents in areas with higher PM_2.5_ concentrations recognizing the effect of air pollution on themselves as more dangerous before the interventions (see the ‘Intervention effects by segment’ section).

## Discussion

To show that messages that remind respondents that they are supported by their relatives can moderate attitudes to recognizing a risk source as dangerous in the real world, we compared three different messages (CG, TG1, and TG2). The message for TG2 (main target group) significantly moderated the subjective assessment to recognize air pollution as dangerous for future generations (main target variable), compared with the control group, CG.

In the initial status before the interventions, the respondents who recognized air pollution caused by industrialization as more dangerous were young women. This tendency was observed for the effect of air pollution on both future generations and the respondents themselves. The perception that air pollution is dangerous was stronger for future generations than for the respondents themselves. The intervention effect in TG2, which used both textual and visual information that highlighted the support from older generations and of future generations, is clearly stronger for future generations (Future generations) than for the respondents themselves (Yourself). While the TG2 effect for future generations was significantly larger than both the TG1 and CG effects (Future generations), the TG2 effect for the respondents themselves was significantly larger than that for TG1, although it was not significantly different from that of CG (Yourself). Thus, considering the intervention effects for attitudes toward both future generations and the respondents themselves, the message created for TG2 should be employed.

Although there is a difference between TG1 and TG2 in the way that the information is presented, TG1 and TG2 includes essentially the same information. Nevertheless, the additional illustration for TG2 significantly increases the perception that the respondents are being supported by the older generations, and has a larger effect in moderating the attitude to perceiving air pollution as dangerous for both future generations and themselves, compared with TG1.

One interpretation could be that the effect arises from the search cost [23] or information overload [24], which is the idea that processing information or decision-making requires mental or cognitive resources, and thus for information provision to be accepted, the presentation should be as concise as possible. The textual information for TG1 (and TG2) might be too long to read, which could decrease the intervention effect that the message originally had. In fact, in the open-ended question there are respondents who felt the TG1 message should be more casual (see the ‘Qualitative survey’ section). Presenting the illustration in TG2 may help to convey the information in an easier way, by reducing the search cost of reading the textual information, which is a problem when the textual information is presented alone. Another interpretation could be that the illustration for TG2 works as an advert picture, which improves attitudes toward adverts [25]. In our case, the DID effect for future generations in TG1 is positive yet statistically insignificant, whereas the DID effect in TG2 is stronger and significant. This increase in the DID effect might be caused by stimulating the visual processing of the respondents, boosting their understanding about the cycle of benefits inherited from a generation and passed to the next generation.

In terms of a nudge with salience, important factors to draw attention are novelty, simplicity, and relevance to who read the provided information [3]. The intervention effect of our designed message in TG2 could be explained in this context too. The visual information comprising a set of colorful illustration materials gives contrast to the monotonous textual messages and hence can give an impression to be novel. Furthermore, it is easy to understand and simple compared to the lengthy textual messages. These novelty and simplicity of the visual part could boost sense of the relevance to the respondents, in that they are supported by their older generations and are supporting their future generations. The largest intervention effect in TG2 might be eventually achieved upon all these factors for salience improved by the additional illustration.

The analysis of our internet questionnaires suggests that well-designed information can moderate the perception of air pollution caused by industrialization as dangerous.

## Conclusion

We conducted an RCT using internet-based questionnaires to ascertain the intervention effect of the designed messages to moderate attitudes to perceive air pollution as dangerous. We compared three groups that received different messages. The first control group received only a basic textual message about the positive points of industrialization and negative points of air pollution caused by industrialization. The second group received the message that the control group received, with additional textual information describing industrialization as a benefit from older generations and also to future generations. The third group received the message that the second group received, with additional visual information to highlight the supportive benefits among generations.

The combination of textual and visual information significantly increased the respondents’ perception that they are supported by their older relatives, including parents and grandparents, compared with the other two types of information provision. The presentation of the information worked as a nudge and produced significant increase in moderating the perception of air pollution as dangerous for future generations, compared with the control group. The respondents that showed a higher intervention effect were younger women and had higher agreeableness.

Although this paper focused on the risks of air pollution caused by industrialization, the idea that stimulating the perception of being supported by relatives enhances attitudes related to altruism could be used for various applications. One such example is promoting energy conservation behaviors. Whereas most of the previous experiments investigated the effects of messages that stimulate social norms by informing people how well other people are conserving energy, other types of representation could be applied and could even be more effective. In future work, we will investigate how our designed messages can be used for other forms of information provision.

We have shown that information provision with salience as a nudge can be designed based on evolutionary insights. According to the MINDSPACE categorization there remain 8 types of nudges aside from salience, where evolutionary methodologies could potentially be useful. Thus, other future work will be reinterpretation of the other nudge types to investigate how evolutionary theories can be applied to develop unexplored nudges.

## Supporting information

S1 Data PlosOne (CSV)

S1 Questionnaire (DOCX)
