# Supplementary material for: Designing information provision to serve as a reminder of altruistic benefits: A case study of the risks of air pollution caused by industrialization
Source: PLoS One. 2020 Jan 16;15(1):e0227024. doi: 10.1371/journal.pone.0227024 (PMC6964856; doi:10.1371/journal.pone.0227024)
Supplement: S1 Questionnaire — (DOCX) [file pone.0227024.s002.docx]

**Questionnaire**

*Questions for interventions*

- *Q*_pre_

How do you think air pollution caused by industrialization affects the following people in daily life?

|  | **Safe** | **Slightly Safe** | **Neutral** | **Slightly Dangerous** | **Dangerous** |
| --- | --- | --- | --- | --- | --- |
| **Future generations** | 1 | 2 | 3 | 4 | 5 |
| **Yourself** | 1 | 2 | 3 | 4 | 5 |

- *Q*_post_

(One of the messages for CG, TG1, and TG2 is shown.**)**

**[Please read the message about industrialization and air pollution and answer the question.]**

Please answer the question below again. How do you think air pollution caused by industrialization affects the following people in daily life?

|  | **Safe** | **Slightly Safe** | **Neutral** | **Slightly Dangerous** | **Dangerous** |
| --- | --- | --- | --- | --- | --- |
| **Future generations** | 1 | 2 | 3 | 4 | 5 |
| **Yourself** | 1 | 2 | 3 | 4 | 5 |

**S Table. Summary statics for *Q*_pre_ and *Q*_post_ (*n*=2764).**

| **Question** | | **Mean** | **Standard Deviation** | **Min** | **Max** |
| --- | --- | --- | --- | --- | --- |
| ***Q*_pre_** | **Future generations** | 3.87 | 0.98 | 1 | 5 |
|  | **Yourself** | 3.26 | 1.04 | 1 | 5 |
| ***Q*_post_** | **Future generations** | 3.69 | 1.03 | 1 | 5 |
|  | **Yourself** | 3.27 | 0.98 | 1 | 5 |

*Other questions*

- *Q*1

(One of the messages for CG, TG1, and TG2 is shown.**)**

Upon reading the message above, do you think you are receiving benefits that increase your health and quality of everyday life from your older relatives, including parents or grandparents?

| **I am benefitting** | **I am benefitting slightly** | **I am not benefitting much** | **I am not**  **benefitting** |
| --- | --- | --- | --- |
| 1 | 2 | 3 | 4 |

- *Q*2

(One of the messages for CG, TG1, and TG2 is shown.**)**

Upon reading the message above, do you think industrialization is giving benefits of increasing health and quality of everyday life to your younger relatives, including children or grandchildren?

| **It has benefits** | **It has some benefits** | **It has few benefits** | **It does not**  **have benefits** |
| --- | --- | --- | --- |
| 1 | 2 | 3 | 4 |

- *Q*3

(One of the messages for CG, TG1, and TG2 is shown.**)**

Please let us know your impressions or thoughts when you read the above message.

- *Q*4

Please answer each of the questions below.

#Please answer about your children.

#Please answer 0 for all the questions if you have no children now.

1. How many children do you have?
2. How many children are living with you?
3. How many children are working in a paid job?

- *Q*5

Please let us know your parents’ current status about their living and working. (Please check as many as necessary for each question)

|  | **Father** | **Mother** | **None** |
| --- | --- | --- | --- |
| 1. **Living with you in the same house (or at the same site)** |  |  |  |
| 1. **Working in a paid job** |  |  |  |
